# Exemplifying an archetypal thorium-EPS complexation by novel thoriotolerant *Providencia thoriotolerans* AM3

**DOI:** 10.1038/s41598-021-82863-4

**Published:** 2021-02-04

**Authors:** Arpit Shukla, Paritosh Parmar, Dweipayan Goswami, Baldev Patel, Meenu Saraf

**Affiliations:** 1 Department of Microbiology and Biotechnology, University School of Sciences, Gujarat University, Ahmedabad, Gujarat 380009 India; 2 Department of Biological Sciences and Biotechnology, Indian Institute of Advanced Research (IIAR), Gandhinagar, Gujarat 382426 India

**Keywords:** Microbiology, Bacteria

## Abstract

It is the acquisition of unique traits that adds to the enigma of microbial capabilities to carry out extraordinary processes. One such ecosystem is the soil exposed to radionuclides, in the vicinity of atomic power stations. With the aim to study thorium (Th) tolerance in the indigenous bacteria of such soil, the bacteria were isolated and screened for maximum thorium tolerance. Out of all, only one strain AM3, found to tolerate extraordinary levels of Th (1500 mg L^−1^), was identified to be belonging to genus *Providencia* and showed maximum genetic similarity with the type strain *P. vermicola* OP1T. This is the first report suggesting any bacteria to tolerate such high Th and we propose to term such microbes as ‘*thoriotolerant*’. The medium composition for cultivating AM3 was optimized using response surface methodology (RSM) which also led to an improvement in its Th-tolerance capabilities by 23%. AM3 was found to be a good producer of EPS and hence one component study was also employed for its optimization. Moreover, the EPS produced by the strain showed interaction with Th, which was deduced by Fourier Transform Infrared (FTIR) spectroscopy.

## Introduction

The aftermaths of atomic bombings of Hiroshima and Nagasaki (1945), more than 2000 nuclear tests (1945–2017), the Chernobyl nuclear power plant disaster (1986) and more recently, the Fukushima Daiichi nuclear disaster (2011), highlight the release of considerable radioactive waste (radwaste) to the environment use of various radionuclides has led to the creation of considerable radioactive waste (radwaste). Similar, but relatively in very miniscule scale, all the nuclear power plants, among other industrial, medical, or technological activities, tend to generate radwaste globally. Consequently, nuclear power plants are responsible for generation of about 95% of the radioactivity worldwide^1^. Microbes, being the most primitive lifeforms on earth, tend to adapt rapidly to such ionizing radiations. Among the naturally occurring radionuclides—uranium (^238^U), potassium (^40^K), calcium (^41^Ca) and radon (^226^Ra); thorium (^232^Th) is one of the most abundant naturally occurring radionuclide (NOR), especially in parts of South Indian coastal line of Tamil Nadu and Karnataka^2^. Based on the radioactivity, composition and phase states of radwaste, they have been categorized into 3: high-level waste (HLW), intermediate-level waste (ILW) and low-level waste (LLW) waste^3^. ILW and LLW are currently being disposed using one of the following procedures: precipitation, ion exchange, evaporation, and reverse osmosis, either singly or in tandem. ILW is carefully disposed in soil at an intermediate depth, while near-land surface disposal is preferred for LLW. This increases the probability of contaminating the soil and in turn exposing its microflora to minute levels of gamma-radiation. For disposal of liquid radwaste, they are concentrated and conditioned, followed by its immobilization in cement, polymer or other highly durable matrix^4,5^. HLW are placed into nuclear waste repositories which are located deep underground^6^.

Conventional methods lack specificity, have special requirements, and are costly which is why, a much cleaner biological approach is being investigated for the remediation of radwaste. Microorganisms, being survivor of various calamities on earth over the time, are being exploited in remediation of radwaste owing to their advantages of being environment friendly and easy to grow, in addition to being amenable and adaptable while retaining their self-sustenance with generation of no or minimal polluting by-products^7^. Bacterial resistance to radionuclides has intrigued researchers globally^2,8^. In recent years, studies pertaining to the devastating Chernobyl accident has led to the discovery of a distinctive phenomenon of ‘*radiostimulation*’, where *Cryptococcus neoformans*, a fungus, showed preference of a radiotrophic mode of nutrition over the conventional chemotrophism^9–11^. Such radiotrophic is yet to be reported in bacteria.

**Figure 1:**
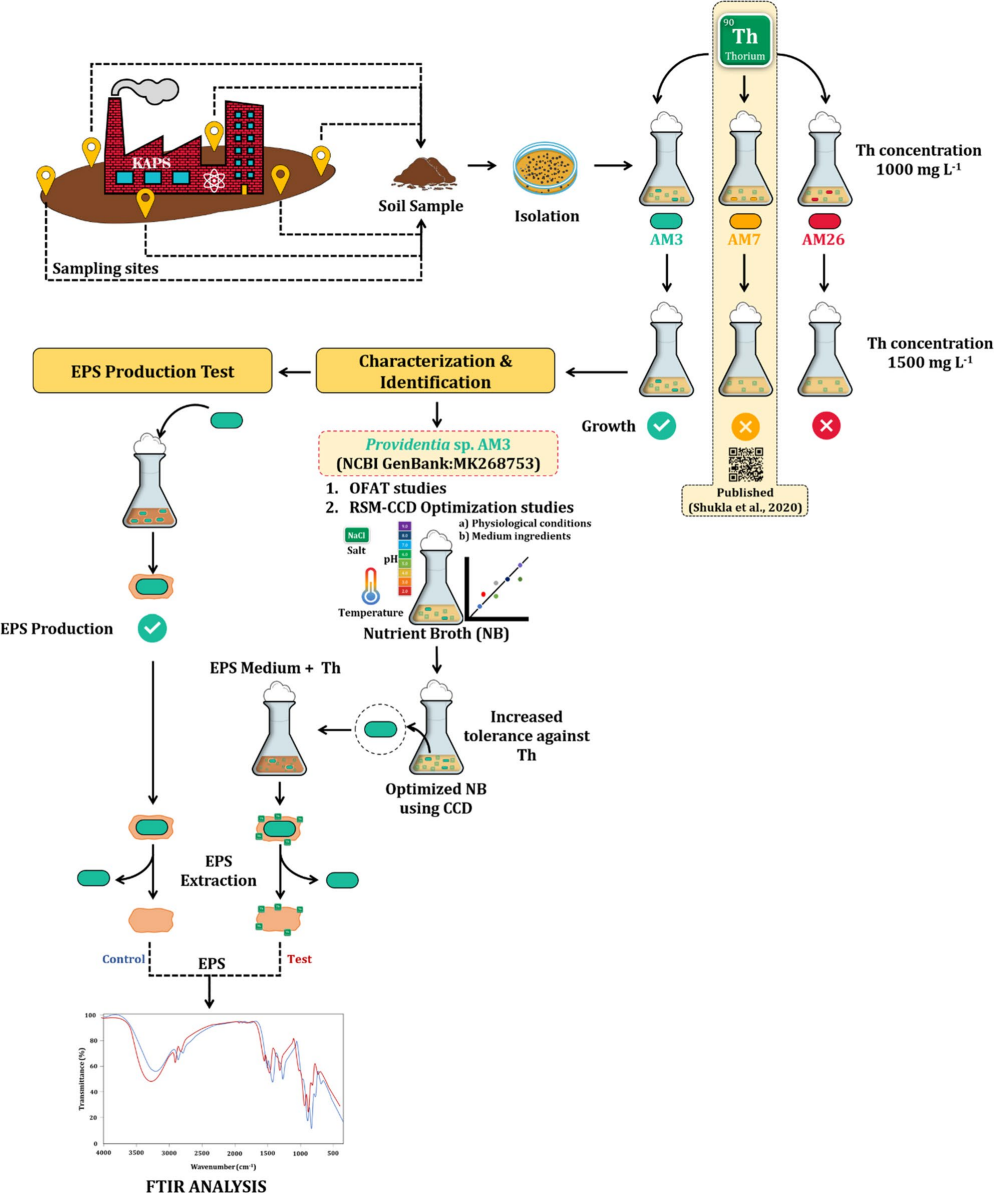
Schematic workflow of the presented work (image created by PP in MS Office 365 ProPlus, PowerPoint).

Thorium is a significant radionuclide since it represents a vital part of a nuclear fuel cycle^12^. The development of Th-powered atomic power stations has led to a surge in the use of Th, thereby necessitating the development of strategies to curb and remediate Th-rich radwaste. Investigating microbes that possess capabilities to withstand and remediate Th is, therefore, the need of the hour. Therefore, the rationale of the present study is to cultivate the indigenous bacterial flora of the soil sample collected from the vicinity of an atomic power station. The cultivated bacteria were subjected to high concentrations of Th (1000–1500 mg L^−1^) and the bacterium exhibiting the most thorium resistance, strain AM3 was selected for further studies. A schematic representation of the presented work is shown in Fig. 1. The outcomes of this study have significant repercussions for developing efficient and eco-friendly strategies for microbial remediation of radionuclides.

## Materials and methods

### Isolation, screening and bacteria cultivation

A composite soil sample was collected from the outer periphery of Kakrapar Atomic Power Station (KAPS), Surat (Gujarat, India). The station is located at 21° 13′ 58.8″ N 73° 22′ 01.8″ E and soil samples were collected during summer-monsoon transitional period in July 2017. Briefly, the samples were aseptically collected from six different sites which were mixed in situ to obtain maximum microbial soil diversity followed by isolation of thorium tolerant bacteria. The soil composition was provided in a previous study^13^. Thorium tolerance assay was performed by supplementing thorium nitrate AR [Th(NO_3_)_4_·5H_2_O] in the culture medium within the range of 100 and 1500 mg L^−1^ and the growth was determined by turbidimetric assay using spectrophotometer. Of all the strains isolated, gram-negative strain AM3 showed maximum tolerance to thorium thus, leading to its selection for further studies. The strain AM3 was cultivated in nutrient broth (NB, HiMedia MM244, India) for 20–24 h at 30 ± 2 °C. Colony of AM3 on nutrient agar (NA) medium appeared as a translucent dew drop with no pigmentation, along with a typical ‘*ropy*’ consistency, strongly suggesting its exopolysaccharide forming capability. In microscopic assessment, AM3 appeared as short rods and during gram-staining, it retained the counterstain safranin. The culture was maintained on nutrient medium at 4 °C and, prior to any study, cells were activated by inoculating a loop-full of culture in fresh medium, following an incubation at 30 ± 2 °C up to 18–22 h with agitation at 150 ± 5 rpm. The activated culture, with an optical density of 0.9 ± 0.1 at 600 nm, was used as inoculum for further studies. The incubation conditions, used for activation of the culture, were kept constant for subsequent studies, unless stated otherwise. The response (growth) was determined spectrophotometrically at 600 nm.

### ICP-OES analysis of soil

For Inductively Coupled Plasma-Optical Emission Spectroscopy (ICP-OES) analysis, the soil was digested using triacid method of the soil^14^. Briefly, 15 mL of triacid [HNO_3_:H_2_SO_4_ and HClO_4_ = 9:2:1] was added to 5 mL of raw soil sample. The mixture was then kept on hot plate (100–200 °C) for 15 min. It was then allowed to cool to room temperature and the final volume was made to 100 mL. The concoction was then passed through the filter (twice) before being analyzed. Twenty-one reference standard solutions of the metals were used for their detection in soil.

### 16S rRNA gene analysis

**Figure 2:**
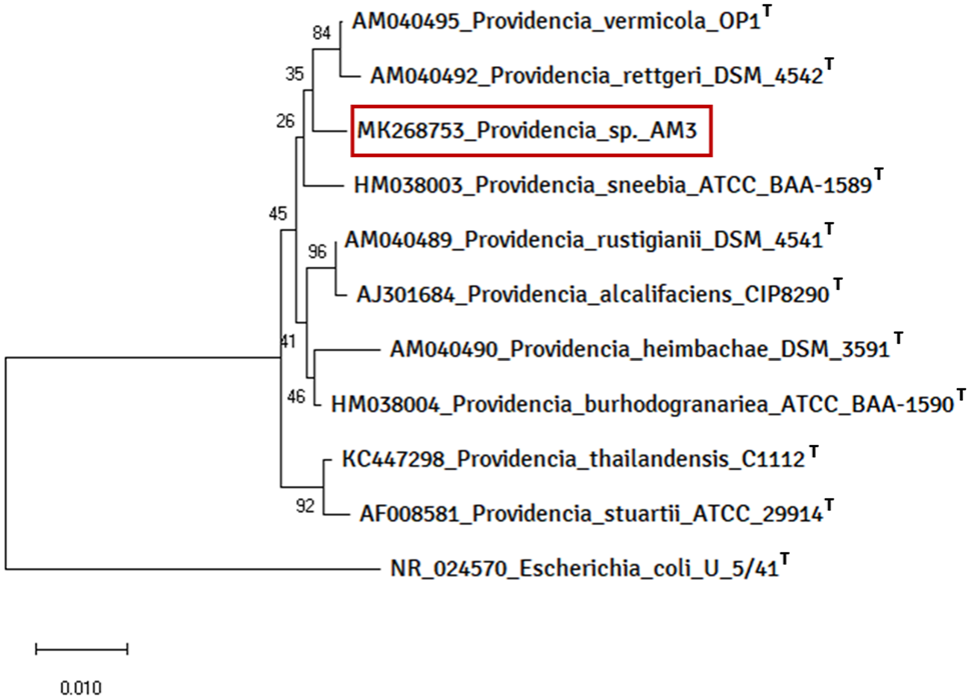
Phylogenetic tree of strain *Providencia* sp. nov. AM3 including all nine member species of *Providencia* genus. *Escherichia coli* serves as an outgroup. ^*T*^*, Type species *(image created in MEGA X)^17^*.*

Genomic DNA of AM3 was extracted as per HiMedia HiPurA Bacterial Genomic DNA Purification Kit (HiMedia-MB505, India) and the 16S rRNA gene was amplified universal primers SRB-255 (5′ GAGAGTTTGATCCTGGCT 3′) and SRB-257 (5′ ACGGCTACCTTGTTACGACTT 3′)^13^. The amplicons were sequenced by Sanger’s dideoxy method and the sequence so obtained was aligned with similar sequences in GenBank—using NCBI BLASTn algorithm for its molecular identification^15,16^. The genus was identified based on the maximum similarity of already existing sequences in the database. After identification, the sequence was submitted to GenBank. For phylogenetic analysis, the 16S rRNA sequences of the type strains belonging to *Providencia* genus were retrieved and all the sequences, along with the sequence of AM3, were aligned using ClustalW algorithm in the software MEGA X^17^. The alignment data was used to construct Neighbour-Joining phylogenetic tree with the bootstrap value of 1000 replications (Fig. 2). The revealed lineage in the phylogenetic tree, was used to determine AM3′s closest relative from the type strains belonging to the genus *Providencia*.

### Optima growth factors

**Figure 3:**
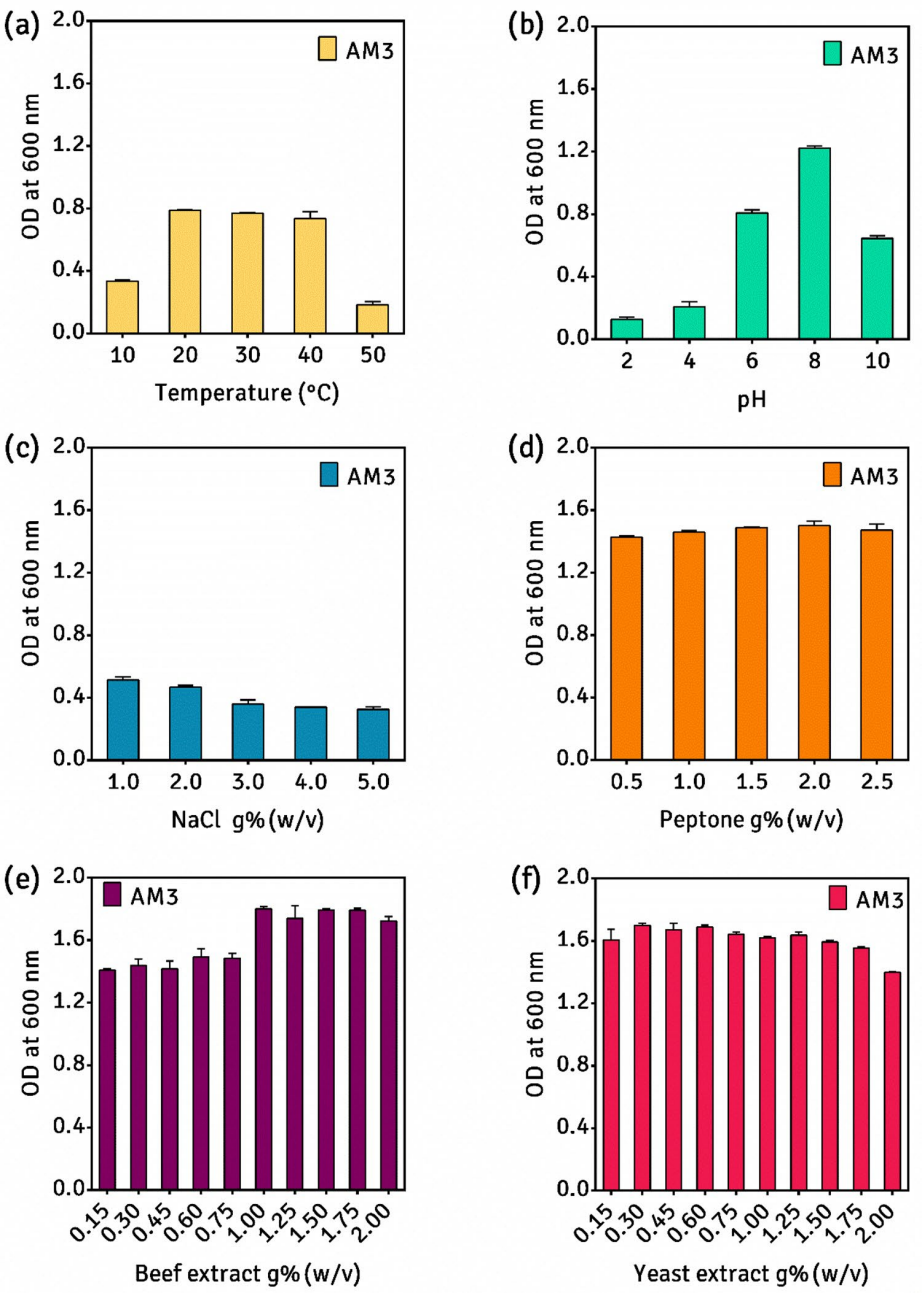
Effect of physical parameters: (**a**) pH and (**b**) temperature and results of one-factor study: (**c**) NaCl, (**d**) peptone, (**e**) yeast extract, (**f**) beef extract on growth of *P. thoriotolerans* sp. nov. AM3 (image created in GraphPad Prism 6)*.*

It is imperative to investigate the factors affecting cell growth before optimizing the medium components for maximum growth. For the determination of the optimum temperature to identify whether the microbe is psychrophilic, mesophilic, or thermophilic, the inoculated broths were incubated at five different temperatures: 10, 20, 30, 40 and 50 (± 2 °C), and pH 7. For the determination of the optimum pH, we adjust the pH of the inoculated broths to five different values (2, 4, 6, 8 and 10) and incubated them at the optimum temperature (Fig. 3). The pH of NB was adjusted using 0.1 M H_2_SO_4_ and 0.1 M NaOH. For optimization of the medium components, the effect of each of the four components of NB on growth of AM3 was determined by varying the concentration of the test ingredient individually, while keeping others constant^18^. The study was performed in triplicates and their mean with *standard deviation* (SD) is plotted.

### Response surface methodology (RSM)

**Figure 4:**
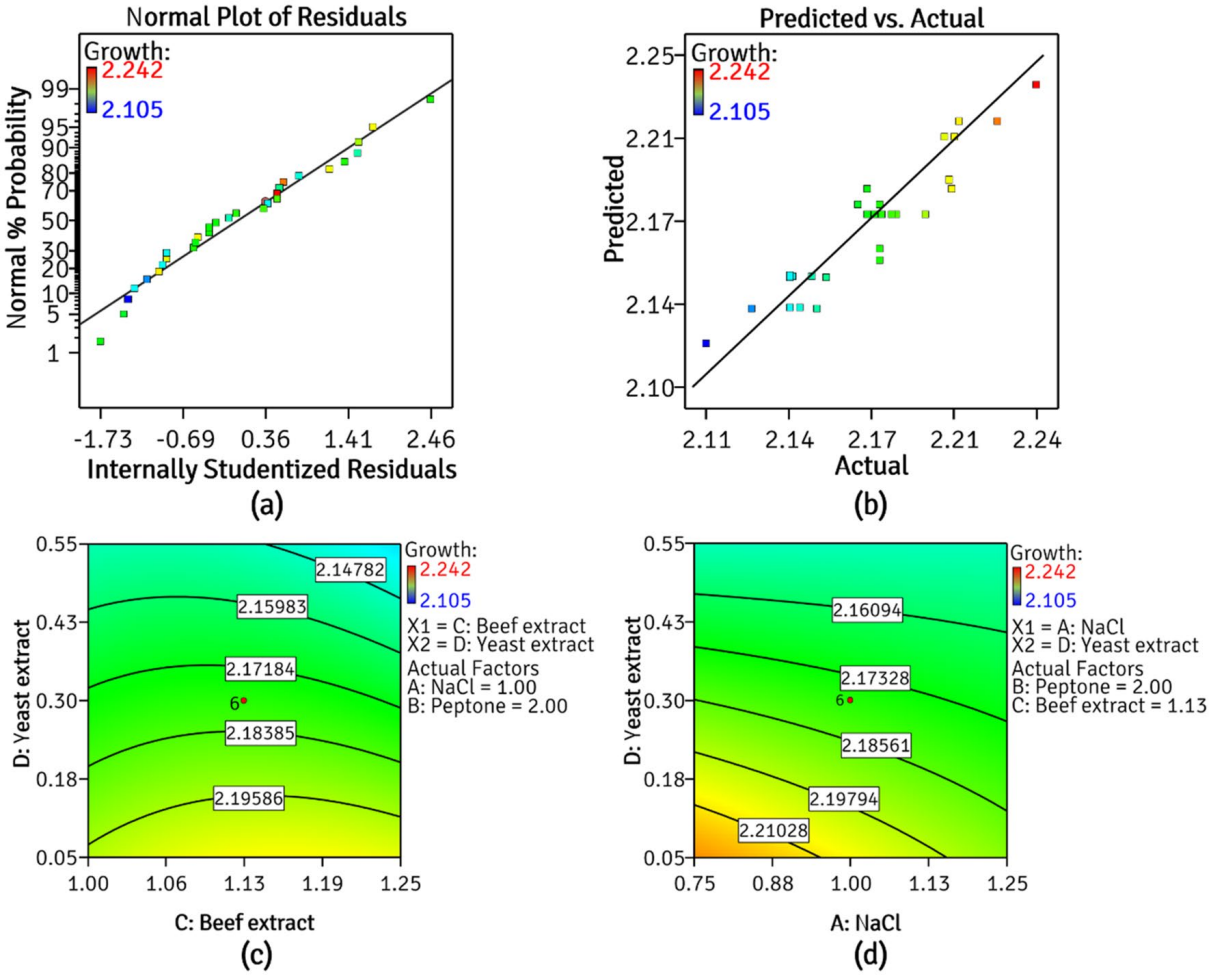
CCD results (**a**) normal probability plot (**b**) predicted vs actual plot (**c**) and (**d**) contour plots depicting significant interactions of NB components on growth of *P. thoriotolerans* AM3 (image created in Design-Expert v7.0).

The *RSM CCD* (Central Composite Design) experiments were designed using the optima growth parameters found in the previous experiments as centre points for all the four components of the NB: beef extract, peptone, yeast extract and sodium chloride (Table 1). The four medium components used for *CCD* were assumed to be *independent variables* and were considered as *numeric factors* in designing the experiments. The *steepest ascent point* (maximal point) of the variable from one component study was considered as *centre point* for optimization. In summary, *RSM CCD* consisted of a 30-run block that included six centre points (Table 2). The physical variables: incubation time, pH and temperature were fixed at 18–22 h, 7.0 ± 0.3 and 28 ± 2 °C, respectively. The response (growth) was measured spectrophotometrically, in terms of absorbance at 600 nm. Stat-Ease Design Expert version 7.0 (Stat-Ease Inc., Minneapolis, USA) was used for statistical designing and analysis of *RSM CCD* experiments. The tools and techniques used for statistical analysis were chosen to determine the adequacy of the model to navigate its successful optimization (Fig. 4). Statistical analyses included; (1) *the analysis of variance (ANOVA)*, (2) *studentized F test* and (3) graphical representations such as *Box–Cox plot*, *Cook’s distance*; in addition to *backward elimination regression* of model terms for *point prediction*^19,20^.

**Table 1:** Variables and their levels for CCD.

Sr. no.	Variables in g% w/v	Coded term	Levels				
			− 2	− 1	0	+ 1	+ 2
01	NaCl	A	0.50	0.75	1.00	1.25	1.50
02	Peptone	B	1.50	1.75	2.00	2.25	2.50
03	Beef extract	C	0.875	1.00	1.125	1.25	1.375
04	Yeast extract	D	0.00	0.05	0.35	0.55	0.8

**Table 2:** Central composite design.

Std. no.	(A) NaCl	(B) Peptone	(C) Beef extract	(D) Yeast extract
	g% w/v			
1	1.00	2.00	1.13	0.80
2	1.00	2.00	1.13	0.30
3	1.00	2.00	1.13	0.00
4	1.25	1.75	1.00	0.05
5	1.25	1.75	1.00	0.55
6	0.75	1.75	1.00	0.55
7	1.00	2.00	1.13	0.30
8	1.25	1.75	1.25	0.55
9	0.75	2.25	1.00	0.05
10	1.00	1.50	1.13	0.30
11	1.25	2.25	1.00	0.05
12	1.25	2.25	1.00	0.55
13	1.00	2.00	0.88	0.30
14	1.00	2.00	1.13	0.30
15	1.00	2.00	1.13	0.30
16	0.75	1.75	1.25	0.05
17	0.50	2.00	1.13	0.30
18	1.00	2.50	1.13	0.30
19	1.00	2.00	1.13	0.30
20	0.75	1.75	1.00	0.05
21	0.75	2.25	1.00	0.55
22	0.75	2.25	1.25	0.55
23	1.00	2.00	1.38	0.30
24	0.75	2.25	1.25	0.05
25	1.00	2.00	1.13	0.30
26	1.50	2.00	1.13	0.30
27	1.25	2.25	1.25	0.05
28	0.75	1.75	1.25	0.55
29	1.25	1.75	1.25	0.05
30	1.25	2.25	1.25	0.55

### Growth analysis

**Figure 5:**
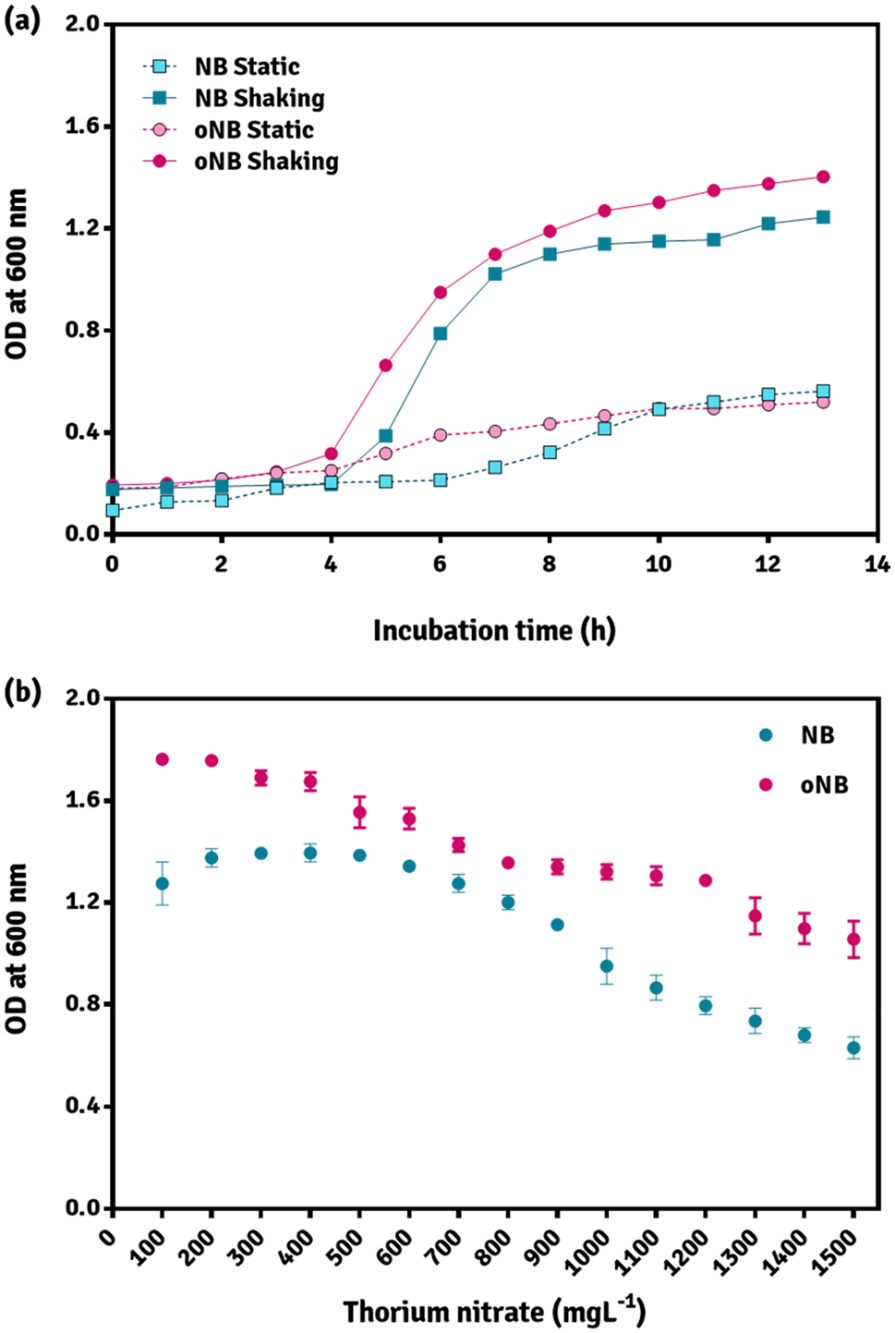
(**a**) Comparison of growth of P. thoriotolerans sp. nov. AM3 on NB and optNB in static and shaking conditions (**b**) Tolerance to thorium nitrate by AM3. *OD 1 corresponds to 10^9^ CFU/mL of P. thoriotolerans sp. nov. AM3 (image created in GraphPad Prism 6).

The difference in the growth rate of AM3 was determined by turbidimetric assay using spectrophotometer by allowing the culture to growth in both the medium-nutrient medium (NM) and optimized nutrient medium (oNM) under optimized physical variables. The samples were withdrawn from the media at an interval of every 30 min after inoculation and the turbidimetric assessment was performed over the period of 14 h-till the stationary phase was achieved. Generation time was determined as per Braga and colleagues^21^. Following to this growth assessment, the impact of Th on growth of AM3 in oNM was compared with NM (Fig. 5). The rationale here, is to minimize intrinsic factors affecting the growth which allows accurate determination of tolerance of Th by AM3.

### EPS production and recovery

**Figure 6:**
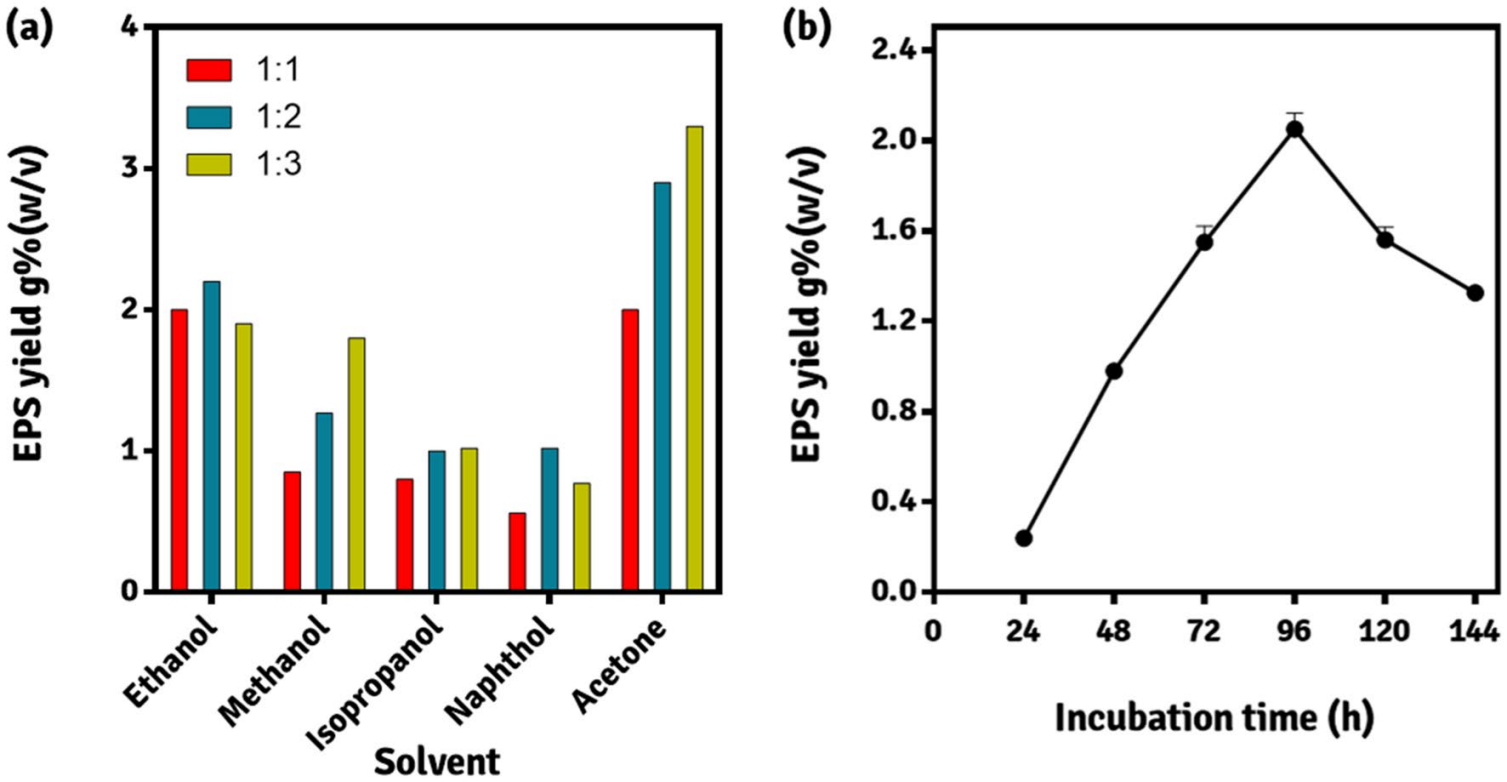
EPS study (**a**) efficiency of extraction solvent for recovery of EPS. 1:1, 1:2 and 1:3 indicate the ratio (v/v) of EPS broth to the solvent used (**b**) effect of incubation time on EPS production. (Image created in GraphPad Prism 6).

The medium for production of EPS (MPE) was used to determine the EPS producing capability of AM3. The medium components of MPE are (in g %); sucrose 4.0, yeast extract 0.5, casein hydrolysate 1.5, K_2_HPO_4_ 1.0, NaCl 0.25 and sodium acetate 1.2; with the pH adjusted to 7.0 ± 0.05. The details for selecting this media for EPS production are presented by Shukla and colleagues^16,18^. Sucrose was added aseptically to the fermentation broth, after sterilization^22^. Production flasks were inoculated with 5% v/v inoculum of actively growing culture and the EPS production was assessed after 144 h of growth at 30 ± 2 °C under agitation of ~ 150 rpm on an orbital shaker. The production broth was centrifuged at 10,000 rpm for 20 min at 4 °C and three volumes of chilled acetone was added to the recovered pellet for precipitation and recovery of capsular EPS. The extracted EPS was recovered using centrifugation and was allowed to dry to a constant weight for its quantification. The EPS production is represented in terms of g % w/v^23^. The recovery of EPS was optimized using various solvents in different concentrations, 1:1, 1:2 and 1:3 v/v (Fig. 6).

### One component study of EPS production medium

Effect of incubation time on EPS production was studied by withdrawing samples aseptically every 24 h for 144 h (Fig. 6). In addition, each of the six ingredients of MPE medium was varied at at-least five levels (Table 3). The effect of each component of MPE on EPS production was determined by varying the concentration of the test ingredient at a time, individually, while keeping others constant^18^.

**Table 3:** Results of effect of EPS medium components on EPS production.

Sr. no	Variable under study	EPS yield g % w/v	pH	Viscosity (mPa s)	Flocculating activity (%)	Total carbohydrate content g % w/v	Total protein content g % w/v
01	**Sucrose** **g% w/v**						
	04	03.34	8.0	01.92	84	02.37	0.04
	08	06.28	7.5	02.08	70	05.92	0.06
	12	05.16	7.0	02.87	50	04.66	0.07
	16	03.58	8.5	02.25	30	02.21	0.08
	20	02.92	9.0	02.43	25	01.92	0.06
02	**Yeast extract** **g% w/v**						
	0.5	01.98	8.0	01.92	84	00.87	0.31
	1.0	02.22	8.7	01.85	74	01.46	0.68
	1.5	02.30	8.0	01.90	71	01.66	0.87
	2.0	02.92	8.0	01.91	64	01.31	0.33
	2.5	03.10	8.0	01.91	63	01.50	0.73
	3.5	03.30	6.5	01.13	59	01.19	0.15
	4.5	04.03	7.3	01.16	64	01.62	0.20
03	**Casein hydrolysate** **g% w/v**						
	1.5	03.94	8.0	01.92	84	02.87	0.40
	3.0	08.14	8.0	01.90	10	06.95	0.10
	4.5	07.46	8.0	01.99	28	07.37	0.02
	6.0	05.28	7.0	01.02	17	03.97	0.02
	7.5	04.90	8.0	01.07	43	02.57	0.04
04	**K**_**2**_**HPO**_**4**_ **g% w/v**						
	1.0	01.14	8.0	01.92	84	00.87	00.03
	2.0	01.98	9.0	01.93	79	01.23	00.01
	3.0	03.35	9.0	01.97	78	02.60	00.01
	4.0	03.84	8.0	01.96	63	03.06	00.01
	5.0	06.17	8.7	01.98	70	05.95	00.02
	7.0	06.68	7.0	02.54	79	05.23	00.05
	9.0	07.94	7.0	02.51	53	06.34	00.04
05	**NaCl** **g% w/v**						
	0.25	03.98	8.0	01.92	84	00.87	00.36
	0.50	05.68	8.5	02.02	30	02.95	00.40
	0.75	05.46	9.0	02.98	30	04.87	00.50
	1.00	05.28	7.0	02.98	32	04.26	00.54
	1.25	04.00	7.0	02.98	30	01.00	00.39
06	**CH**_**3**_**COONa** **g% w/v**						
	1.2	01.98	8.0	01.92	84	01.33	00.03
	2.4	02.18	9.0	02.19	32	01.90	00.04
	3.6	02.40	7.0	02.44	33	01.96	00.03
	4.8	02.18	9.0	02.14	25	01.47	00.01
	6.0	01.82	9.0	02.06	33	01.15	00.05

### EPS characterization

**Figure 7:**
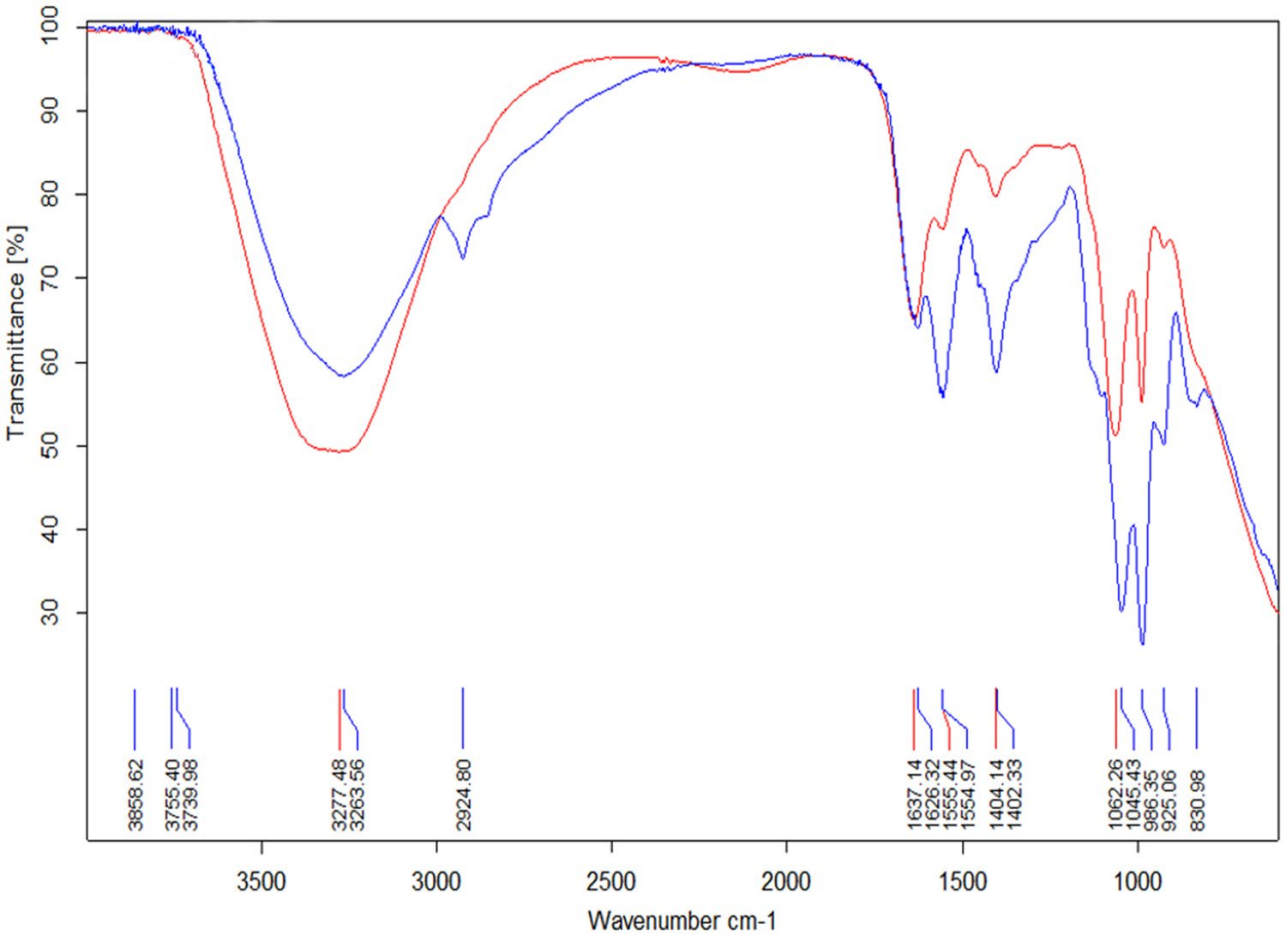
FTIR analysis of *P. thoriotolerans* sp. nov. AM3 EPS (blue) and EPS^Th+^ (red) (image created in OPUS, Bruker Alpha).

The dried EPS extract was dissolved in double-distilled water for its characterization through the determination of total carbohydrate (TC) and protein contents by the phenol–sulfuric acid and Lowry’s methods, respectively^24,25^. This was performed to determine the presence of proteoglycans in the EPS. The viscosity of both the production MPE broth and the extracted EPS was measured by Brookfield DVI-Prime viscometer (Brookfield Engineering Laboratories, USA). EPS was characterized for its flocculating activity^26^ and its Th adsorption was determined by FTIR spectroscopy; Bruker, ALPHA-ATR system, Bruker Optics GmbH, Ettlingen, Germany; was used with 4 cm^−1^ resolutions and 16 scans in the range of 600 to 4000 cm^−1^ for FTIR (Fig. 7). Thorium scavenging activity by the EPS was determined by allowing the culture to grow in MPE medium supplemented with Th (1500 mg L^−1^) and this medium was designated as MPE^Th+^. The EPS extracted from this culture broth, designated as EPS^Th+^ was analyzed using FTIR spectroscopy and the spectra of both—EPS and EPS^Th+^, were compared to determine the interaction of Th with the functional groups of EPS.

## Results and discussion

### Isolation, screening and bacteria cultivation

The results of ICP-OES analysis of the soil are previously described^13^. Of the elements detected, significant amount of Fe was detected followed by Ti and Mg whereas, Sb and Sr were barely within the detectable limit. Fe and Mg act as cofactor for enzymes and their high concentrations indicates active microbial metabolism in soil. A relatively high amount of Ti is astounding since it has been proven to be inhibitory to microbial growth^27^.

Strain AM3 showed exceptional tolerance to Th (1500 mg L^−1^) and could still thrive with 50% cell density at 1500 mg L^−1^ when compared to cell densities under non-stress condition. Strain AM3 exhibited high tolerance to Th and was thus, screened for further studies (Fig. 5). Recently, the means of heavy metal and radionuclide interactions with microorganisms have been put forth which mainly attributes the resistance against such toxic elements, to the presence of S-layer, glycocalyx, slime, and biofilm production by the candidate microbe^2^. Moreover, the bacterium which is reported to withstand the maximum ionizing radiations of 10^4^ Sv, *Deinococcus radiodurans*, is a gram-positive tertacocci that has acted as the flag-bearer of ‘*radioresistance*’ that is exhibited among bacteria^28^. The mechanism of radioresistance in *D. radiodurans* has been assigned largely, to the multiple copies of its genome which upon exposure to radiation is able to revert the inflicted damages. Among the gram-negative bacterial spectrum, the class gammaproteobacteria outweighs other classes and its members, belonging to the genera *Photobacterium*, *Serratia* and *Pseudomonas* stand out in terms of degree of radioresistance^15,18^. Therefore, an objective of the present study also included the biochemical characterization and evaluation of Th-tolerant microbes.

### Thoriotolerant microorganisms

The screened strain AM3 showed 15 times greater tolerance to Th than previously ever reported by any bacteria and hence would be recognized as a novel Th-tolerant microbe. We had previously reported *Ochrobactrum intermedium* AM7 to survive in a medium supplemented with 1000 mg L^−1^ Th and the strain under present (AM3) study showed ~ 50% better tolerance to Th^18^. Moreover, there are reports suggesting *Arthrobacter* sp. J001 and *Bacillus* sp. J003 to thrive at 100 mg L^−1^ of Th in the culture media and were categorized as Th-resistant microbes^13,28^.

The word ‘*thoriotolerant*’ (thôr’ēô + ˈtɑlərənt) is derived from the radionuclide, ‘*thorium*’ and the Latin participle ‘*tolerans*’*. Thorium* (thôr'ēəm) is named after the Scandanavian god ‘*Thor*’ whereas, *tolerant *(ˈtɑlərənt) signifies ‘to tolerate’. We propose to term a microbe as ‘*thoriotolerant’* when it is able to thrive and tolerate thorium above 1000 mg L^−1^.

### Molecular identification

16S rRNA partial gene sequence was used for the identification of AM3. BLASTn algorithm showed AM3 to be 96.71% similar to type strain *Providencia vermicola* type strain OP1 and hence, the sequence was submitted as *P. vermicola* AM3 to GenBank (accession no. MK268753). The type material identity with *P. vermicola* type strain OP1 was found also to be 96.71%, in addition to its EZcloudBio status of 95.43% strongly distinguishes strain AM3 as a novel species. We, therefore, propose the name *Providencia thoriotolerans* sp. nov..

### *Providencia thoriotolerans* sp. nov.

We had previously reported *Ochrobactrum intermedium* AM7^18^, which was able to tolerate Th up to 1000 mg L^−1^. To the best of our knowledge, this is the first study to demonstrate such high levels of Th tolerance by any bacteria, by far exceeding 500 mg L^−1^ Th as compared to *O. intermedium* AM7. *Providencia thoriotolerans* (*thoriotolerans:* thôr’ēô+ˈtɒlərəns) is named after the microbe’s exceptional tolerance to thorium. *Providencia* genus belongs to *Enterobacteriaceae* family of *Enterobacteriales* order of *Gammaproteobacteria* class, comprising of nine species of gram-negative rods^16^. Some strains of *P. vermicola* have been successfully incorporated in sequestration and biosorption of palladium, lead sulphite, and platinum^29–31^. Here, we report for the first time, the isolation of *thoriotolerant P. thoriotolerans* sp. nov. type strain AM3. The phylogenetic tree (Fig. 2) shows an intraspecies similarity of AM3 with the type strains of *P. vermicola* (OP1), *P. rettgeri* (DSM 4542) and *P. sneebia* (ATCC BAA1589).

### Optima growth factors

The growth parameters including, physical as well as chemical factors were checked. Impact of temperature on growth depicted that, the culture showed better growth in the mesophilic range, where the growth was observed in the temperature ranging from 20 to 40 °C and the optimum temperature range falling between 20 and 30 °C (Fig. 3a). A dramatic decrease in growth was obtained at the temperatures above 40 °C with about ~ 75% decline in growth at 50 °C when compared with the optimum growth temperature. The reports suggest that several members of *Providencia* thrive well at human body temperature^32^.

As for the impact of different pH on the growth of AM3, the bacterium failed to grow in the acidic range, specifically below six, and the strain showed a preference to grow in a slightly alkaline environment in the pH range (8 ± 1). At pH above ten, the growth declined by 50% when compared with the optimum pH (Fig. 3b).

Certain strains of *Providencia* sp. are reported to colonize on catheter by its ability to produce biofilm and have known to cause infections in immuno-compromised patients^32^. So, it is inevitable to determine its pathogenicity by assessing β-hemolytic activity on blood-agar^33^. The strain was tested negative for haemolytic activity. Out of several strains of *Providencia*, only *P. stuartii, P. rettgeri, P. alcalifaciens, P. rustigianii and P. heimbachae* are reported to be uro-pathogenic and not all the strains of this genus is claimed to be pathogenic^34^.

Among the physical growth parameters, pH and temperature are recognized as the variables that directly correlate with alteration in the pore size of the cellular membrane thereby, playing a major role in imparting resistance to metals, salts, and ions^35^. A proton-rich, acidic pH is known to dissolve majority of salts and cations. A poor acid-tolerance by *P. thoriotolerans* AM3, within a narrow range of overall neutral-pH, led to the assumption of the strain to be highly unlikely candidate to tolerate high concentration of metals or radionuclides^36^. However, on the contrary, the strain was found to be highly tolerant to Th under narrow pH range (7–9). For this phenomenon, we assume that the strain might have acquired resistance in the soil that is already rich in metals and radionuclides that may have led to its exceptional Th-tolerance^37^. The solubility of metals and salts depends largely on temperature as the higher temperatures may lead to precipitation or salting-out of metals. Owing to such salting-out phenomena, several salts tend to reprecipitate after a successful sterilization cycle and therefore, a special procedure for metal related studies must be followed^38^.

The chemical characterization of one-factor study (Fig. 3f) suggests that, from the nitrogen sources supplemented in NB, the requirement of beef extract was found to be fulfilled at 1.0 g% whereas, peptone exhibited maximum growth at 2.0 g%. No significant decline in growth was observed by either, increasing (up to 2.5%) or decreasing (up to 0.5%) peptone concentrations. The growth had decreased by 10% and ~ 30% when the concentration of sodium chloride was increased to 2 and 3 g%, respectively. Notably, the concentrations of neither of the nitrogen sources radically affected the growth of AM3. Based on the findings, for AM3, we recommend a medium comprising (in g% w/v): 1.00, 2.00, 0.30 and 1.00 of beef extract, peptone, yeast extract and NaCl, respectively. These values were, therefore, considered as centre points (0-level) for constructing the CCD (Tables 1, 2).

The physical and chemical requirements for fulfiling the basic growth necessities of AM3 were studied by one component assay. The results of this study will pave a path to design the experiments involving RSM and hence, one component study was performed prior to the construction of CCD^26,29,30^. If the factors included in CCD are non-significant for growth, it may give false results and designs in CCD and so one-component study was performed to nullify such extraneous errors^38^.

### Response surface methodology (RSM)

*RSM* is a statistical tool which is employed to study the interaction among variables for the desired response and to identify and optimize the significant factors. Here, the desired response is the growth of *P. thoriotolerans* AM3. *CCD* of experiments provide prediction models to navigate through each variable under study, while also informing the significant level of a variable that may be responsible for the obtained response. Figure 4a–d depicts the interactions among variables in the present study. For successful optimization of variables, a *point prediction* of the model is achieved by a thorough analysis of the responses and their experimental validation^39^. A *backward elimination regression* with *alpha out* was applied for fitting of *mean*, *linear*, *2FI*, *quadratic*, and *cubic* process models. The *linear model* was suggested by the software showing the prediction of optimal values (Table 4). There was no interaction among individual medium components and only peptone was found to be *insignificant* for the optimization process.

**Table 4:** ANOVA for response surface reduced quadratic model with backward elimination regression.

Source	Sum of squares	Degrees of freedom	Mean square	F value	p value
A-NaCl	1.442E−003	1	1.442E−003	10.05	0.0043**
C-beef extract	7.350E−005	1	7.350E−005	0.51	0.4814^NS^
D-yeast extract	0.021	1	0.021	143.10	< 0.0001**
AD	9.000E−004	1	9.000E−004	6.27	0.0198**
CD	4.410E−004	1	4.410E−004	3.07	0.0929^NS^
C^2^	1.056E−003	1	1.056E−003	7.36	0.0124**
Residual	3.300E−003	23	1.435E−004		
Lack of fit	2.939E−003	18	1.633E−004	2.26	0.1866^NS^
Pure error	3.608E−004	5	7.217E−005		
Cor total	0.028	29			
Model	0.024	6	4.074E−003	28.39	< 0.0001**

*p > 0.05, **p > 0.01, *NS* non-significant.

Non-significant terms removed via backward regression alpha to exit = 0.100.

R^2^ = 0.8811, Adj-R^2^ = 0.8500, Pred-R^2^ = 0.7324.

To achieve the maximum growth of AM3, *RSM-CCD* was employed for the statistical designing of experiments to determine the optimum concentrations of NB ingredients. For the *quadratic model* suggested by the software, *one-way ANOVA (F-test)* for growth was evaluated (Table 4). Equation (1) shows the effect of sodium chloride (*A*), peptone (*B*), beef extract (*C*) and yeast extract (*D*) on predicted growth of AM3 (*Y*) where, the negative and positive signs are indicative of the antagonistic and synergistic effect of the variables on growth^40^.

1 $${\text{Y}} = {2}.{18} - {7}.{75}0{\text{E}} - 00{\text{3A}} - {1}.{75}0{\text{E}} - 00{\text{3C}} - 0.0{\text{29D}} + {7}.{5}00{\text{E}} - 00{\text{3AD}} - {5}.{25}0{\text{E}} - 00{\text{3CD}} - {6}.0{\text{56E}} - 00{\text{3C}}^{{2}}$$

The obtained *determinant coefficient R*^*2*^* value of 0.8811* suggests that, *88.11%* variation in response was due to independent variables. Moreover, the *adjusted-R*^*2*^ and *predicted-R*^*2*^ values were in agreement with one another which strongly implies the correct fitting of the selected polynomial model. *Non-significant lack-of fit* (*p value* = *0.1866*) showed the goodness of model fit. As depicted in Fig. 4a, a straight line in normal probability plot indicate a normal distribution of data. In the parity predicted *vs* actual plot (Fig. 4b), the data points were obtained near central straight line indicating close proximity between the actual experimental results (represented by dots) and that predicted by the software (represented by a straight line) thereby, confirming the validity of the obtained results as well as the *fit of the model* (Design Expert, Stat-Ease Inc., Minneapolis, USA)^18^.

Based on the statistical significance (p values), model terms A, D, AD and C^2^, were found to be significant, while the interactions between A, C and D had no effect on the response. Backward elimination regression was used to fit responses to the quadratic model which led to elimination of the non-significant terms. The one-way ANOVA results suggest that in NB, peptone or beef extract had no significant effect on the growth of AM3 (Table 4). Therefore, variables A and D were identified as constricting components and even a slight variation in their concentration influences the growth of AM3 significantly. The significant interactions between NaCl and yeast extract are shown as response plots (Fig. 4c,d) where, NaCl negatively affected the response upon its interaction with yeast extract.

The optimization of media and production processes in biology employ RSM-CCD due to its statistical rigor^41,42^. Basically, RSM-CCD construct a model to assess cumulative effect of cause (variable) and its effect (response)^43^.

### Growth analyses

Aeration condition was preferred by AM3, as its rapid growth was observed in oxygen rich conditions when contrasted with the static condition, suggesting aerophilic nature of the strain (Fig. 5a). The growth study revealed that, in aerated conditions, the length of the lag phase of AM3 was 4 h followed by the logarithmic phase of 6 h. The growth, estimated by the optical density, was found to stabilize after 10 of incubation which represent the stationary phase of the cells. The generation time of *P. thoriotolerans* AM3 was improved to 48 min in optM (optimized NB^Th+^ medium supplemented with 1500 mg L^−1^ of Th), as opposed to 54 min in NB which validated the findings of the RSM-CCD study. Furthermore, a reduction of 6 min in growth rate was a result of successful point prediction and optimization of significant variables of NB. Biological systems have a tendency to directly undergo indigenous metabolic and physiological modifications when their nutrition is varied thereby, affecting growth of the organism^44^.

To determine the resistance to Th, *P. thoriotolerans* AM3 was exposed to an optM^Th+^. As can be inferred from Fig. 5b, Th-tolerance by AM3 was substantially improved in optM^Th+^ with a slight increase in its biomass. The isolate was able to resist and thrive at 1500 mg L^−1^ of Th, which is distinct from the results obtained in NB^Th+^ medium which may be attributed to an increase in nitrogen content of the medium^8^. Also, the extraneous factors, which may have a negative effect on the growth, were corrected during the optimization process making supplementation of Th; the only stress to the bacterium in optM^Th+^.

### EPS assessment

#### One component study

The viscosity of the medium is affected by the diffusion of EPS produced by the microbe into the production medium. There exist several methods to extract and recover the EPS from the medium: using salts, detergents, organic acids, and solvents. Previous reports have suggested that organic solvents are the preferred agents in order to rapidly precipitate the EPS without inflicting any damage to its composing glycosidic linkages. For extraction and recovery of EPS, the extraction solvent and its corresponding volume to be added to the production broth was studied (Fig. 6a). Acetone and methanol could be the preferred solvents to extract EPS from the production broth by maintaining their ratios at 1:1 (v/v) w.r.t the culture broth. At 1:2 ratio (broth:solvent) the efficiency of ethanol, isopropyl alcohol and naphthol peaked, whereas maximum efficiency of methanol and acetone was observed at 1:3 ratio (broth:solvent). The efficiency of ethanol and naphthol decreased when their concentration increased above two-volumes to that of production broth. Methanol, isopropyl alcohol and acetone demonstrated an increase in extraction efficiency with an increase in the broth to solvent ratio, 1:3 > 1:2 > 1:1. The results are in agreement with our previous study^18^. For any extraction process, the partition coefficient is responsible for the determination of the amount of solvent to be used^45–47^. However, the amount of solvent to be used for extraction is inversely proportional to its partition coefficient.

Three volumes of acetone in one volume of production broth proved to be the apparent choice, as the extraction solvent for recovery of EPS produced by *P. thoriotolerans* AM3. In our findings, acetone even at its lowest volume was still able to recover the maximum EPS as compared to the higher volumes of other solvents. Morevoer, FTIR spectroscopy suggested that acetone had minimal deterimental effect on the cross linking of sugars in EPS^26^. Therefore, for extraction of EPS, acetone was used for further studies and its FTIR assessment was also performed.

Figure 6b depicts the yield of EPS over an incubation period of 144 h. The maximum yield was obtained after 96 h of incubation, where the increase in EPS yield from 48 to 72 h and 72 to 96 h was 50 and 33%, respectively. This indicates a gradual increase in EPS production, typical for secondary metabolites. After 120 and 144 h, the EPS yield was found to decrease by 25 and 38%, respectively. This could be due to the depletion of the medium components and the exhaustion of sugar content in the production medium. The components of EPS (carbohydrate and protein) could be biodegraded by the cell during conditions of nutrient paucity. Our results are in agreement with that of Ye and colleagues^48^. Therefore, after the EPS one-factor study, an incubation time of 96 h was set.

#### EPS characterization

As shown in Table 3, sucrose, casein hydrolysate, yeast extract and K_2_HPO_4_ were identified as significant components of the production medium. Upon increasing the concentration of sucrose from 4 to 8 g%, an increase of 88% in EPS yield was observed. Similarly, 3 g% casein hydrolysate and 9 g% K_2_HPO_4_ were found to be favorable for maximum EPS production, whereas a steady increment in the yield was observed with the increase in concentration of yeast extract. No substantial decrease in production was observed when the concentration of NaCl and CH_3_COONa were increased. In terms of viscosity, sucrose and NaCl were the major contributors. The maximum viscosity of 2.98 mPa s was observed at 0.75% NaCl, and the minimum of 1.85 mPa s at 1.0% concentration of yeast extract. After incubation, there was no significant change in the pH of the production medium indicating negligible role of the medium components in dictating the medium pH. K_2_HPO_4_ was found to significantly affect the flocculating activity of EPS.

When *P. thoriotolerans* AM3 was allowed to grow in MPE^Th+^, the EPS^Th+^ yield obtained was similar to the production medium that was devoid of Th. Furthermore, no change in the viscosity and flocculation activity of the EPS was observed, which suggests that the yield of EPS was not affected by Th supplementation in the media. The latter may be due to the unaltered C:N ratio (upon addition of Th). Moreover, Th may not have interacted with any critical enzymes or central metabolic pathways that govern the production of EPS^49^. The EPS is physically the result of cross linking charged residues of sugars, that may interact with Th present in the medium. This causes the adsorption of Th on to the cross linking of EPS, which can be investigated using FTIR spectroscopy^18,50^.

FTIR is a highly efficient and sophisticated technique for investigation of metal interaction with EPS. There are several research articles solely using FTIR to evaluate the interacation of metals with EPS^46^. FTIR analysis can predict the adsorption and the ionic interaction that the metal atoms can make with the EPS^47^. In the general opinion, the complete disappearance or significance in the peak intensity represents the ionic interactions while the shifting of the peaks without any changes in the peak intensity show adsorption^48–50^. The fingerprint region of FTIR spectra falling below the region of 1500 cm^−1^ is considered to be critical for the functional groups, and specifically for EPS, this region shows the presence of –C–O carboxyl groups, –C=O carbonyl stretching, –N–H amide groups of amino, –P–O bond formation and phosphate of phosphorylated sugars^51–56^. The FTIR spectra of the unloaded control (EPS) and loaded (EPS^Th+^) EPS of *P. thoriotolerans* AM3 were measured in the range of 4000–600 cm^−1^ (Fig. 6).

The detailed analysis of the FTIR spectra involving adsorption and ionic interaction of Th with EPS is explained as follows: characteristic broad and strong peaks obtained at 3263 cm^−1^ for EPS and 3277 cm^−1^ for EPS^Th+^, indicate the –O–H hydroxyl stretching to shift in the wavenumber with an increase in the absorption intensity by 20%, further suggesting its strong binding with the –OH group of sugars found in EPS. The peak of EPS at 2924 cm^−1^ suggests extending of –C–O carboxyl bonds, while the disappearance of this peak in the spectra of EPS^Th+^ demonstrates the interaction of Th with the carboxyl group, which is found at the last carbon of the monosaccharide repeating units of EPS^53^. The small peaks ranging from 2900 to 3000 cm^−1^ in the EPS spectra indicate the behaviour of alkyl groups by the stretching of –CH, –CH_2_ and –CH_3_. The disappearance of these peaks in the spectra of EPS^Th+^, suggests the stabilizing of alkyl groups by adsorption of Th. Similarly, there are reports where such phenomena are depicted by the adsorption of metals with EPS^53,54^. The signature –C–O carboxyl group peaks in the fingerprint region (1400–1500 cm^−1^) were prominent in EPS, while the peak intensity had decreased to half in EPS^Th+^, thereby reassuring the binding of Th with these functional groups^55^. The peak intensities were greatly varied when the FTIR spectra of EPS and EPS^Th+^ were compared and it extended to the fingerprint region ranging from 2000 to 700 cm^−1^. The most significant change was observed to the peak at 1555 cm^−1^ (for EPS), which showed a very strong intensity, but the same peak had vanished in EPS^Th+^, with a residual hump, suggesting strong interaction of Th with –C=O carbonyl stretching as well as –N–H amide groups of amino sugars or proteins of EPS. All these changes in the spectra strongly indicate the ionic interaction of Th with EPS. The peak at 1626 cm^−1^ for EPS shifted to 1637 cm^−1^ in EPS^Th+^, indicating the binding of Th with the amide of the –CO–NH peptide bond. Similar phenomenon is also found when Cu forms a complex with the peptide bond in the Biuret reaction^57^. Similar studies were also performed for EPS extracted from *Pantoea agglomerans* with positively charged Hg, Cu, Ag, As, Pb, Cr, and Cd while *Exiguobacterium* UE1 and UE4 interacting with Cr (VI) showed similar interaction of metal with the amide, amine, carboxyl and hydroxyl group of EPS^54–56,58^. The peaks in the range of 960–1100 cm^−1^ indicate ionic –P–O bond formation while the signature peaks falling within 1000–1100 cm^−1^ also represent carboxyl group^21^. For the spectra of EPS, strong pair of peaks at 925 and 1045 cm^−1^ might indicate the presence of phosphate and carboxyl group. These peaks for EPS^Th+^ had shifted signifcantly, changing its value to 986 and 1062 cm^−1^, indicating interaction of Th with phosphorylated sugar moeities present in the EPS. Positively charged Th may interact with the negatively charged functional groups of the EPS by making ionic interactions. This phenomenon may help in trapping Th in the matrix of EPS and thereby, assisting its adsorption. Hence it can be deduced that aliphatic functional groups aid in sorption of Th by the EPS with adsorption, while the functional groups such as carboxyl groups, amides, and phosphates moeities of sugars found in the EPS tend to make ionic interactions with Th.

## Conclusions

Radwaste is primarily disposed of by burial in deep geological repositories. The resistance to tolerate radionuclides, acquired by microbes residing in such niches, can be seen as potential candidates to remediate radwaste. Studies pertaining to assess microbial tolerance to radionuclides is scarce but can pave a path for identifying novel strains possessing tolerance to radwaste. To address this blind spot, we cultivated the indigenous bacterial flora from the soil in the vicinity of an atomic power station with an objective to study strains that show tolerance to radionuclides. We have isolated and identified a strain (AM3) that showed exceptional tolerance to Th up to 1500 mg L^−1^. By far, such high tolerance to Th is not yet reported and we propose to use the new term ‘*thoriotolerant’* for the microbes exhibiting tolerance to Th beyond 1000 mgL^−1^. The outcome of one component study was used for the construction of RSM CCD of experiments to identify and optimize the variables affecting growth of AM3. The 16S rRNA partial gene sequence analyses of AM3 suggested the strain to be novel and is proposed to be named as, *P. thoriotolerans* sp. nov.. Moreover, the strain showed exceptional EPS production even in presence of Th and the EPS so produced also possessed the ability to absorb and remove Th, which was determined using FTIR spectroscopy. Such microbes pose a tremendous scope to devise a strategy for microbial-mediated remediation of radwaste.

## Data Availability

The 16S rRNA partial gene sequence of *Providencia* sp. AM3 is submitted to NCBI GenBank database with accession number MK268753 (https://www.ncbi.nlm.nih.gov/nuccore/MK268753.1/).
